# Mentorship Programs for Faculty Development in Academic General Pediatric Divisions

**DOI:** 10.1155/2011/538616

**Published:** 2011-11-17

**Authors:** Jennifer Takagishi, Sharon Dabrow

**Affiliations:** Division of General Pediatrics, Department of Pediatrics, University of South Florida, Tampa, FL 33606, USA

## Abstract

*Introduction*. Mentoring relationships have been shown to support academicians in areas of research, work/life balance, and promotion. *Methods*. General pediatric division chiefs accessed an electronic survey asking about mentorship relationships, their ability to create a mentorship program, and resources needed. *Results*. Dyadic mentorship programs were available at 53% of divisions. Peer mentorship programs were available at 27% of divisions. Overall, 84% of chiefs believed that dyadic mentorship would benefit their faculty. 91% of chiefs believed that peer mentorship would benefit their faculty. Chiefs were interested in starting peer (57%) or dyadic (55%) mentorship programs. Few divisions had a peer mentorship program available, whereas 24% already had a dyadic program. 43% of chiefs felt that they had the tools to start a program. Many tools are needed to create a program. *Discussion*. General pediatric division chiefs acknowledge the benefits of mentoring relationships, and some have programs in place. Many need tools to create them. Pediatric societies could facilitate this critical area of professional development.

## 1. Introduction

Dyadic, traditional senior to junior mentorship, relationships have been shown to have both career and psychosocial functions and usually proceed through several stages before often concluding [1]. Peer or collaborative mentorship relationships, a partnership with members of the same approximate age and/or career stage, also go through phases along a continuum of forms of relationships. However, they may last far longer and be perceived differently dependent on the individuals' career stage [2]. Collegiality may be a central reason to maintain these relationships, in addition to the effects on career. However, some of these relationships may actually turn out to be negative influencers [3].

Mentorship in academic medicine has been noted to be “important” by about 2/3 of clinical faculty, but only about 1/4 of faculty receive formal mentoring [4]. Having a mentor, or preferably multiple mentors, is strongly related to satisfaction with mentoring and overall job satisfaction. Satisfaction with mentoring also was associated with greater job satisfaction and less expectation of leaving the institution within subsequent years [5]. Mentorship has been reported to be an important influence on personal development, career guidance, career choice, and productivity. Mentoring can have an important effect on research productivity, including publication and grant success [6]. Lack of mentoring has been cited as the first or second most important factor hindering faculty career progress in academic medicine [7].

Reviews of medical school students [8], faculty [9], pediatric residents [10], and physicians [11–13] have shown varied manners to provide mentorship to these groups, both in traditional dyadic and peer-group settings. More recently, mentorship groups have geared specifically to junior faculty of either gender [14–16], women faculty [17–19] and underrepresented minority faculty, which may or may not include women as a distinct subgroup [20–22]. These groups have shown success in increased productivity, satisfaction, and an improved sense of “fit” or empowerment. Particularly for women and underrepresented minority faculty, peer-group mentorship has been lauded as necessary due to a lack of senior mentors who represent these groups and may act as role models. Women have found it more difficult to find senior role models, possibly due to a different construct for seeking out mentorship and a different work model [3, 6, 17, 19], although this has not been found by all authors [5]. 

Basic science faculty may be better able to find mentors than clinical faculty, although this has not been found consistently [4]. In fact, mentoring was found to be more prevalent among faculty in the tenure track than among clinician-educator or research track faculty members [5]. Clinician-educators may be at increased risk for inadequate mentorship for reasons such as having been ill prepared to define their academic roles in preparation for promotion, objective criteria for success as educators have not been well defined and outlined, and the promotions process at many universities may not recognize the scholarly achievements of clinician-educators. Lastly, mentors in medical education may have been few or difficult to find [23].

The need for mentorship, particularly among women and minorities in pediatrics, will continue to grow. In 2009, 30 percent of postresidency pediatricians were minorities and 54.5% were female. More strikingly, in residency, 72.2 percent of residents were female [24]. In contrast, as of 2007, women represented only 14 percent of tenured college of medicine faculty and 12 percent of full professors overall [25], although in pediatric departments, 19 percent of women had achieved the rank of full professor [26].

While some faculty find the career-advancing mentoring they need, others could use help forming and maintain such relationships. Within academic general pediatric divisions, no known study has been undertaken to determine the quantity and support of mentorship opportunities for division members. The objective of this study, therefore, is to assess the availability of mentorship programs, the types of available programs, and to determine the value division chiefs place upon these programs.

## 2. Materials and Methods

A survey (Peer mentorship questionnaire) was designed for general pediatric division chiefs to learn about their dyadic and peer mentorship experiences. It also investigated demographics and mentorship opportunities in their divisions, departments, and colleges of medicine.

The survey was uploaded to the Academic Pediatric Association's (APA) general listserv along with an explanatory letter inviting division chiefs to participate in the study by accessing a link to the survey. In the explanatory letter, the dyadic mentoring model was defined as “the more traditional junior-senior person relationship.” The peer mentorship model was “also called the collaborative mentoring model. In this, mentors may be of the same approximate age and/or stage of career as one another.” No further definitions were given to allow for a broad range of mentorship programs to be reported by the division chiefs. Informed consent was presumed by completion of the survey. Responses were not associated with any identifying data. The University of South Florida Institutional Review Board approved this project. 

The number of general or division chief members on the APA listserv when the survey was sent is unknown, although the number of division chiefs on a separate listserv is 133. The survey was posted on the general listserv so that it could be forwarded by APA members to division chiefs who may not be on the division chief listserv.

## 3. Results and Discussion

Fifty-eight surveys were completed. The gender split was almost equal with 29 (52%) men and 27 (48%) women respondents. The majority were between 50 and 60 years old (*N* = 29, 51%) and held senior rank of professor (*N* = 30, 54%). Regarding the size of the division based on the number of MDs, it was split with 15 respondents (26%) having 0–10, 16 (28%) having 11–20, 16 (28%) having 21–30, and 11 (19%) with over 30 physicians.

### 3.1. Dyadic Mentorship Program

Thirty one (54%) had a dyadic mentorship program at the division level, 26 (45%) at the departmental level and 17 (29%) at the college level. For those that had a program at the College of Medicine, all (100%, *N* = 17) stated that it was available to junior faculty, 12 to midcareer faculty (70%), and only 4 to senior faculty (24%). For the 25 that had a departmental program, 25 (100%) said it was open to junior faculty, 16 (64%) to midcareer faculty, and 7 (28%) to senior faculty. Of the 31 that had a divisional program, 30 (97%) were offered to junior faculty and 20 (65%) offered to midcareer faculty. 

 The majority of division chiefs' (*N* = 44, 76%) responded that they formed a dyadic mentorship relationship with one or more faculty members. Surprisingly, these divisions chiefs often mentor faculty outside their division (*N* = 22, 50%). Forty-eight (84%) thought a dyadic mentorship program would be beneficial for a variety of reasons: assisting with promotion (*N* = 51, 91%), assisting with work-life balance (*N* = 45, 80%), skill development (*N* = 48, 86%), and assistance with research (*N* = 47, 84%).

### 3.2. Peer Mentorship Program

The largest number of respondents had a program at the division level (*N* = 16, 27%), followed by department level (*N* = 11, 19%) and lastly at the College of Medicine (*N* = 6, 10%). At both the department and division levels, 100% of the programs were open to junior faculty. 73% (8) of departmental programs were open to midcareer faculty, versus 87% (13) of the divisional programs. Senior faculty had programs available at 45% (5) of the departments and 67% (10) of the divisions. Thirty-three (57%) said they formed peer mentorship relationship with one or more faculty and the majority (*n* = 19, 58%) said they were outside the division. 49 (91%) stated that it would be beneficial to have a peer mentoring program in the following areas: promotion (*n* = 31, 60%), work-life balance (*N* = 44, 85%), skill development (*N* = 40, 77%), and research (*N* = 39, 75%). 

There was equal interest in creating a peer and/or dyadic mentorship program (*N* = 32 dyadic and *N* = 33 peer). Fourteen (24%) already had a dyadic program whereas only 7 (12%) had a peer program. Twenty-four (43%) stated that they had the tools and knowledge to set up a program, but 18 (32%) were not sure. Items that would be of most help included examples and links to/from other programs (*N* = 11, 78%), and workshops (*N* = 8, 57%). 

 Using the known values for peer and dyadic mentorships and the Chi-square test for determination of independence, a highly statistically significant difference (*P* < 0.001) in the proportion of dyadic versus peer mentorship programs was found at the aggregate level (college plus department plus division). Utilizing the population surveyed, there was a higher proportion of dyadic than peer-based mentorship programs in the academic population overall.

 When comparing the different levels within academia, a statistically significant difference in the higher proportion of dyadic versus peer-based mentorship programs at both the division and department levels was noted, but not at the college level. At the college level, a similar amount of dyadic and peer-based mentorship programs existed.

 To increase the power of the calculations, the above data was reanalyzed with the inclusion of the respondents who did not know whether there was a dyadic- or peer-based mentorship programs available in their academic community. To further bias against the possibility of finding a difference (independence) between the amounts of dyadic versus peer programs, the people who did not know whether there were any programs at all were summed with the people who answered that there were no dyadic or peer programs. The Chi-square tests with the added data confirmed the above conclusions: a greater number of dyadic than peer-based programs in the overall community exist at the department and division levels, but not at the college level.

### 3.3. Limitations

The total number of division chiefs, and the percent who are members of the APA and who are on the listserv, is unknown. Thus, the survey is limited to those division chiefs who either are on the APA general listserv or were forwarded the survey link. Therefore, this survey may over- or under-represent the true percent of divisions, departments, and colleges of medicine with active mentoring opportunities.

Further, not all questions were answered by all the respondents. The statistical analysis compensated for this by assuming that unknown responses were “no” answers, and statistical significance was still obtained at divisional and departmental levels.

Based upon this survey of academic general pediatric division chiefs, both peer and dyadic mentorship programs exist throughout the country with the majority dyadic at the departmental and divisional levels. A possible reason there is no difference in the proportion of dyadic versus peer-based programs at the college level heralds a trend of increasing peer-based programs that has not yet reached the individual departments or divisions of these colleges. The chiefs reported that mentorship programs would benefit their faculty, and many are interested in starting their own programs but may need support such as articles, workshops, and linkages with other programs.

## 4. Conclusions

Given that mentorship programs have been shown to benefit faculty in areas such as promotion, research, and “fit” within the university, the availability of more mentoring programs should benefit academic general pediatricians. Innovative ways to mentor subgroups within academic general pediatrics such as women and hospitalists may be particularly needed. As these innovative programs become more widely available and undergo evaluation to determine their effectiveness, faculty in other specialties within and outside of pediatrics should also benefit.

 Some of these resources already exist but may not be widely known. An internal medicine group has created a program for supporting hospitalist physicians [27] that could be used as a model. The American Association of Medical Colleges maintains a description of several mentoring programs [28]. Several authors have described how to design and support mentoring programs [29–31]. The important role that the mentee plays has also been elucidated [32]. Another potential source for mentors that could be further developed includes more senior physicians specifically taught to guide mentorship in their regions [33].

 The Academic Pediatric Association and Association for Pediatric Program Directors sponsored a Combined Leadership & Peer Mentoring Forum in September 2010. This area of leadership would be an important one for pediatric societies to become more involved in, and ways in which professional societies can assist in career development in academic medicine have been described [34]. Other means to assist could include site linkages to relevant articles, offering opportunities for leaders at successful programs to come to other programs to help them setup, through online or webcast discussions, and workshops at national meetings to help program leaders gain the tools to help. Telephone conferences between sites could also be used along with newer technologies such as videoconferencing and webcasts. 

As the number of mentorship programs increases, future research to assess which type of mentoring—dyadic, peer, or a combination—is best suited for different faculty subpopulations would be a valuable contribution to faculty development programs.

**Table:** 

**Peer mentorship questionnaire.**
*Demographic Information for General Pediatric Division *
*Chiefs*
(1) Gender
Male
Female
(2) Age
30–40 years old
40–50
50–60
60–70
>70
(3) Faculty level:
Professor
Associate Professor
Assistant Professor/Instructor
(4) Size of division (full- and part-time faculty)
— number of MD's
— number of PhD's
— number of ARNP's
*Mentorship Questions—Y=yes, N=no, DK=do not know*
(1) Is there a dyadic mentorship program at the:
College of Medicine level? Y N DK
Departmental level? Y N DK
Divisional level? Y N DK
(2) If so, to whom is it available?
Fellows? Y N DK
Junior faculty? Y N DK
Midcareer faculty? Y N DK
Senior faculty? Y N DK
(3) Outside your role as division chief, have you formed
dyadic mentorship relationship(s) with one or more faculty
members? Y N
(3.a) If yes, are the faculty inside or outside your division?
(4) Do you think a dyadic mentorship program would be
beneficial to your faculty?
(5) If yes, how? Assist with promotion Work-life balance
Skill development
Research Other_____________
(6) Is there a peer mentorship program at the:
College of Medicine level? Y N DK
Departmental level? Y N DK
Divisional level? Y N DK
(7) If so, to whom is it available?
Fellows? Y N DK
Junior faculty? Y N DK
Midcareer faculty? Y N DK
Senior faculty? Y N DK
(8) Outside your role as division chief, have you formed
peer mentorship relationships with one or more faculty
members? Y N
(8.a) If yes, are the faculty inside or outside your division?
(9) Do you think a peer mentoring program would be
beneficial to your faculty? Y N
(10) If yes, how? Assist with Promotion Work-life balance
Skill development
Research Other___________
(11) How interested are you in creating each type of
mentorship program within your division or department?
5=very interested, 1=not at all interested6 already have one
Peer 1 2 3 4 5
Dyadic 1 2 3 4 5
(12) Do you have the tools and/or knowledge to do so? Y N
(13) If not, what tools would be of assistance? Select all
that apply.
Articles Examples from other programs Links to
other programs already developed
Workshop Conference calls Listserv Other
(14) Any other comments?
